# Identifying serum metabolite biomarkers for autoimmune diseases: a two-sample mendelian randomization and meta-analysis

**DOI:** 10.3389/fimmu.2024.1300457

**Published:** 2024-04-15

**Authors:** Wenwen Wang, Manli Huang, Wei Ge, Junling Feng, Xihua Zhang, Chen Li, Ling Wang

**Affiliations:** ^1^ Department of Health Statistics, School of Preventive Medicine, Ministry of Education Key Lab of Hazard Assessment and Control in Special Operational Environment, Fourth Military Medical University, Xi’an, Shaanxi, China; ^2^ Department of Field and Disaster Nursing, Fourth Military Medical University, Xi’an, Shaanxi, China; ^3^ Department of Neurological Intensive Care Rehabilitation, Xi’an International Medical Center Hospital, Xi’an, Shaanxi, China

**Keywords:** autoimmune diseases, biomarkers, Mendelian randomization, meta-analysis, serum metabolites

## Abstract

**Background:**

Extensive evidence suggests a link between alterations in serum metabolite composition and various autoimmune diseases (ADs). Nevertheless, the causal relationship underlying these correlations and their potential utility as dependable biomarkers for early AD detection remain uncertain.

**Objective:**

The objective of this study was to employ a two-sample Mendelian randomization (MR) approach to ascertain the causal relationship between serum metabolites and ADs. Additionally, a meta-analysis incorporating data from diverse samples was conducted to enhance the validation of this causal effect.

**Materials and methods:**

A two-sample MR analysis was performed to investigate the association between 486 human serum metabolites and six prevalent autoimmune diseases: systemic lupus erythematosus (SLE), rheumatoid arthritis (RA), inflammatory bowel disease (IBD), dermatomyositis (DM), type 1 diabetes (T1D), and celiac disease (CeD). The inverse variance weighted (IVW) model was employed as the primary analytical technique for the two-sample MR analysis, aiming to identify blood metabolites linked with autoimmune diseases. Independent outcome samples were utilized for further validation of significant blood metabolites. Additional sensitivity analyses, including heterogeneity test, horizontal pleiotropy test, and retention rate analysis, were conducted. The results from these analyses were subsequently meta-integrated. Finally, metabolic pathway analysis was performed using the KEGG and Small Molecule Pathway Databases (SMPD).

**Results:**

Following the discovery and replication phases, eight metabolites were identified as causally associated with various autoimmune diseases, encompassing five lipid metabolism types: 1-oleoylglycerophosphoethanolamine, 1-arachidonoylglycerophosphoethanolamine, 1-myristoylglycerophosphocholine, arachidonate (20:4 n6), and glycerol. The meta-analysis indicated that three out of these eight metabolites exhibited a protective effect, while the remaining five were designated as pathogenic factors. The robustness of these associations was further confirmed through sensitivity analysis. Moreover, an investigation into metabolic pathways revealed a significant correlation between galactose metabolism and autoimmune diseases.

**Conclusion:**

This study revealed a causal relationship between lipid metabolites and ADs, providing novel insights into the mechanism of AD development mediated by serum metabolites and possible biomarkers for early diagnosis.

## Introduction

Autoimmune diseases (ADs) arise from a disruption in the immune system’s ability to tolerate self-antigens and its subsequent response to these antigens (1). Epidemiological studies have shown a gradual increase in the global prevalence of ADs over the years (2). Individuals with ADs often experience persistent symptoms that significantly impact their quality of life, leading to long-term debilitation, organ dysfunction, reduced work productivity, and substantial medical expenses (3). These challenges not only affect patients but also have a significant impact on the social and economic aspects of society. Despite numerous studies attempting to understand the nature of ADs, their pathogenesis and risk factors remain elusive (4–6). The preclinical phase of AD is characterized by an initial asymptomatic stage of varying duration, followed by non-specific signs and symptoms. Most AD cases have a considerably long prodromal phase with either no symptoms or mild symptoms that can last for several years. Additionally, various inflammatory manifestations may occur and often intensify in the months to years leading up to a clinical diagnosis (7, 8). A promising strategy for preventing ADs could involve addressing adverse lifestyle factors through public health initiatives at the population level, highlighting the importance of effective early screening strategies for ADs.

Controlling inflammation remains a pivotal aspect of ADs therapy, including conditions like RA (9) and SLE (10). A growing body of evidence indicates that immune cell regulatory mechanisms are intricately connected to metabolic pathways, with various subpopulations of immune cells having distinct metabolic requirements that are influenced by disease-specific microenvironments (11–13). For example, effector T cells rely on glycolytic metabolism for their development and function, while regulatory T cells primarily use lipids to generate energy through mitochondrial beta-oxidation, leading to ATP production via oxidative phosphorylation (OXPHOS) (14). Furthermore, inflammatory M1 macrophages predominantly utilize glycolysis, whereas anti-inflammatory M2 macrophages favor beta-oxidation (15). In ADs, the autoinflammatory response is characterized by high energy consumption and involves processes such as adipogenesis, altered glucose metabolism, and glutamine metabolism, resulting in a shift from OXPHOS to glycolysis for energy production. In specific scenarios, such as in RA, hypoxia in the synovial membrane triggers chronic mitochondrial hyperpolarization in T cells, leading to increased glucose metabolism and ATP synthesis (16). Additionally, chronically activated T cells in individuals with SLE and lupus-prone mice exhibit heightened mitochondrial glucose oxidation and hyperpolarization. Individuals with multiple sclerosis (MS) have been found to have elevated plasma levels of acetoacetic acid, acetone, and 3-hydroxybutyric acid, along with altered profiles of circulating lipids and lipoprotein metabolism (17, 18). Additionally, metabolites are compounds that act as intermediates or end products in metabolic pathways. Detecting metabolites in the bloodstream provides a robust method for early disease diagnosis, owing to their accessibility and ease of detection (19). Consequently, understanding the underlying mechanisms linking metabolic changes to AD pathogenesis could lead to the development of new early screening tools and therapeutic interventions (3).

Indeed, while cross-sectional studies have identified associations between blood metabolites and ADs, establishing causality remains a crucial area of investigation. Further research is required to determine if altered metabolite levels are a cause or consequence of ADs. Longitudinal studies incorporating both healthy individuals and those at risk of developing ADs could help elucidate the causal relationships between metabolites and ADs. In addition, interventional studies that examine the effects of lifestyle changes on metabolite levels and disease outcomes may also provide valuable insights into the role of metabolites in ADs. Ultimately, determining the causality of metabolite-AD associations could lead to the identification of novel therapeutic targets and personalized treatment options for individuals with ADs.

The advancement of high-throughput techniques has enabled the simultaneous assessment of a broad range of circulating serum metabolites and genotyping in parallel populations. Genome-wide association studies (GWAS) provide valuable insights into the complex molecular interactions between environmental and genetic factors in disease pathogenesis. Numerous single nucleotide polymorphisms (SNPs) have shown strong associations with serum metabolites. Mendelian randomization (MR), an epidemiological statistical analysis tool, utilizes genetic variation linked to exposure as an unconfounded instrumental variable to assess the relationship between exposure and clinical disease outcomes (20). GWAS have expanded to include metabolic phenotypes, producing maps of genetically determined metabolites (GDM) (21). Several MR causal analyses have explored the link between blood metabolite levels and disease. For example, Huang et al. used MR to identify key pathogenic metabolites influencing both T2D and more severe COVID-19 phenotypes in obese patients (22). Additionally, a study from *Gu* (23) investigated the causal relationship between osteoarthritis at different sites and blood metabolites. Likewise, *Xiao et al.* discovered 11 metabolites with a significant causal association with anxiety disorders (24). Furthermore, *Angela Ge et al.* conducted an MR analysis to examine the causal relationship between serum metabolite composition and MS (25). However, there is still a lack of in-depth research on large-scale blood metabolites and multiple autoimmune diseases.

Therefore, the main aim of this research was to investigate the potential causal relationships between serum metabolites through a two-sample MR and meta-analysis involving six autoimmune diseases: SLE, RA, IBD, DM, T1D, and CeD.

## Materials and methods

### Ethics statement and MR design

This research exclusively relied on publicly available GWAS data, and all necessary ethical approvals, participant consents, and permissions from the original GWAS study are readily accessible. No new data were collected for this investigation, rendering any additional ethical approvals unnecessary.

**Figure 1:**
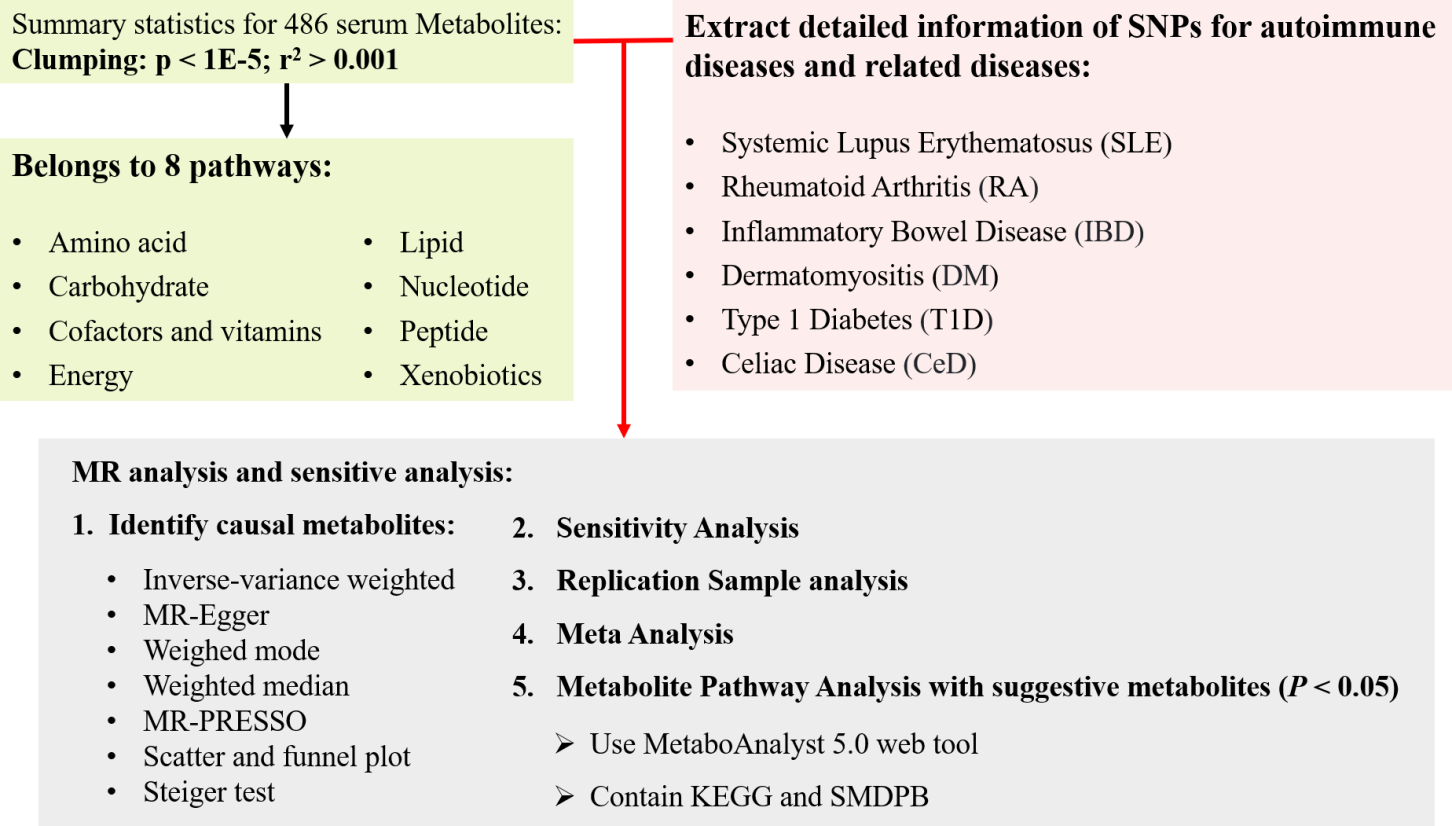
The overview of the research workflow.

The flow chart outlining the methodology of this study is illustrated in **Figure 1**. In summary, serum metabolites function as exposure indicators, while ADs represent the outcomes. Rigorous inclusion and exclusion criteria guided the selection of single nucleotide polymorphisms (SNPs) strongly associated with specific serum metabolites as instrumental variables (IVs). The samples included both discovery and validation cohorts, with significant associations identified through various sensitivity analyses. Furthermore, reverse MR analysis was performed to mitigate potential confounding effects of ADs on the pathogenic serum metabolites.

### Serum metabolites datasets

The metabolomics GWAS server (http://metabolomics.helmholtz-muenchen.de/gwas/) was utilized to obtain genetic association information for serum metabolites. Shin et al. conducted a study involving a total of 7,824 participants from two European population cohorts (21). This cohort consisted of 1,768 participants from the German KORA F4 study and 6,056 from the UK Twin study. Fasting serum samples were subjected to non-targeted mass spectrometry analysis. Following stringent quality control measures, the researchers successfully identified 486 metabolites (comprising 309 known metabolites and 177 unknown metabolites) that demonstrated a genetic influence on serum metabolites. The 309 known metabolites were systematically categorized into eight distinct biochemical classifications, encompassing lipids, peptides, nucleotides, energy, amino acids, cofactors and vitamins, carbohydrates, and exogenous substances.

### Autoimmune disease discovery and validation datasets

During the discovery phase, GWAS summary statistics were obtained from publicly available analyses for each of the six AD types and **Table 1** provides additional information about the dataset. The GWAS meta-analysis for IBD encompassed 12,882 cases of European ancestry and 21,770 controls (26). Furthermore, for RA, the pooled data included information from 1,605 RA cases and 359,589 controls of European ancestry across 18 studies. In the case of SLE, the pooled measurements were derived from a GWAS meta-analysis involving 1,311 SLE cases and 1,783 controls of European ancestry (27). Additionally, summary statistics for T1D were sourced from the discovery phase of the latest GWAS meta-analysis conducted in Finland, involving 1,238 T1D cases and 183,185 controls of European ancestry. Similarly, pooled measurements of DM) were obtained from the GWAS and included 208 DM cases and 213,145 controls of European ancestry. Finally, a pooled genetic measure for CeD was derived from a GWAS meta-analysis that incorporated data from five different sample groups, comprising 4,533 CeD cases and 10,750 controls (28).

**Table 1:** Autoimmune diseases GWAS samples used in this study.

Stage	Trait	Consortium	N.case	N.controls	SNPs
Discovery	IBD	IIBDGC	12,882	21,770	12,716,084
	RA	UK Biobank	1,605	359,589	10,079,899
	SLE	IEU	1,311	1,783	489,877
	T1D	NA	9,266	15,574	12,783,129
	DM	FINNGEN	208	213,145	16,380,451
	CeD	NA	4,533	10,750	523,399
Validation	IBD	NA	25,042	25,042	9,619,016
	RA	UK Biobank	1,401	359,793	9,944,222
	SLE	NA	5,201	9,066	7,071,163
	T1D	FINNGEN	2,542	182,573	16,380,230
	DM	FINNGEN	201	172,834	16,380,281
	CeD	IEU	3,796	8,154	231,359

Furthermore, repeated MR analyses were performed on 486 serum metabolites to facilitate validation of the findings in the discovery database. The validated outcome samples for IBD comprised 25,042 case and 25,042 control samples of European ancestry (29). The validation samples for RA were sourced from the UK Biobank, which included 1,401 cases and 359,793 control samples. Similarly, the local replicate samples for SLE consisted of 7,219 and 15,991 control GWAS of European ancestry (30). Moreover, the duplicate samples for T1D and DM were obtained from the FINNGEN database, which included 5,928 and 201 cases and 183,185 and 172,834 control samples. The GWAS meta-analysis of replicated CeD samples involved 12,041 CeD cases and 12,228 controls of European ancestry (31). More specific information about the data used can be found in **Supplementary Table 1**.

Quality control measures were implemented for SNPs to ensure the validity of the analysis. These measures involved removing non-dual allelic SNPs, SNPs with duplicate rsID or base pair locations, SNPs lacking rsID, SNPs with strand ambiguous alleles, SNPs not present in Phase 3 of the 1000 Genomes Project, SNPs with base pair locations or allele mismatches in Phase 3 of the 1000 Genomes Project, SNPs with interpolated information scores below 0.9, and all SNPs located on the *X* and *Y* chromosomes.

### Selection of instrumental variables

In this MR analysis, the selection of IVs was predicated on three fundamental assumptions. SNPs associated with the metabolite at the genome-wide significance threshold $P<1\times 10^{-5}$ were chosen as potential IVs. Eligible IVs were further refined through a series of quality control steps. Initially, SNPs with inconsistent alleles (e.g., T/C vs. T/G) between the exposure and outcome samples were excluded. Subsequently, palindromic alleles (A/T or G/C) were also excluded. Thirdly, SNPs within each metabolite were subjected to clumping in order to retain only independent variants. The linkage disequilibrium (LD) threshold for clumping was set at ${\text{R}}^{2}<0.001$ within a 500 kilobase distance, utilizing the European-based 1,000 Genome Projects reference panel for estimation. Finally, the MR pleiotropy residual sum and outlier (MR-PRESSO) test was utilized to detect potential horizontal pleiotropy, addressing it by removing outliers (32). To quantitatively ascertain the strength of the selected SNPs as instruments, the ratio of phenotypic variation explained for each metabolite and *F*-statistics of the instrument were calculated (33):


$$F=\frac{r^{2}\left(n-1-k\right)}{\left(1-r^{2}\right)k}$$

Here, $r^{2}\;$ represents the part of the exposure variance explained by IVs, n is the sample size, and k is the number of IVs. A statistic of. signifies a lack of strong evidence for weak instrument bias (34). In this research, IVs with F -statistics less than 10 were classified as weak IVs and were thus excluded. Finally, a coordinated analysis was performed to compare exposure and outcome SNP alleles, excluding the alleles with intermediate effects ( EAF>0.42 ) of palindromic SNPs or SNPs with incompatible alleles (35).

### Mendelian randomization analysis

In this MR analysis, several tests were conducted to evaluate the causal relationship between metabolites and AD. These tests included fixed/random effects inverse variance weighting (IVW) tests (36), weighted median (WM) (37), and MR-Egger (38). The IVW method, known for providing the most accurate estimate for valid SNPs, was utilized as the primary analysis to assess the causal relationship between serum metabolites and ADs, with a significance level set at *P*< 0.05. Additionally, complementary analyses such as WM and MR-Egger were performed to enhance the confidence of the estimates and offer advantages under different assumptions. The WM method yields a consistent causal estimate when at least 50% of the weight comes from valid instrumental variables (37). On the other hand, the MR-Egger regression helps to address pleiotropy when all instrumental variables are invalid (30).

### Sensitivity analysis

The IVW method provides a robust estimate of the causal effect of exposure when all three IV assumptions are met, and it is considered the most reliable MR method. However, if certain instrumental variables contradict the IV assumptions, the analysis may produce erroneous results. Therefore, the following sensitivity analyses were conducted: 1) *Cochrane’s Q* test for IVW and MR-Egger was employed to investigate potential violations of the assumptions due to heterogeneity in the association among individual IVs (36). 2) The intercept estimate of horizontal pleiotropy in MR-Egger was used to assess any independent association of genetic variation with exposure and outcome. 3) Additional MR methods with varying modeling assumptions and strengths were used to enhance the stability and robustness of the results. 4) MR-PRESSO was utilized to identify outliers and correct for horizontal pleiotropy. 5) Individual SNP analysis and leave-one-out (LOO) analysis were employed to evaluate whether individual SNPs influenced the observed associations.

The following principles guided the identification of potentially suitable candidate metabolite IVs associated with ADs: 1) Ensuring amplitude and directional consistency across the results from the four MR analysis methods; 2) Confirming the absence of pleiotropy and heterogeneity; 3) Using LOO analysis to confirm the absence of any influential data points exerting substantial influence on the outcomes.

### Genetic correlation and direction validation

The MR results may potentially generate false positives as a result of genetic correlations between traits (39). Throughout the process of IV selection, SNPs associated with ADs were deliberately excluded. Nonetheless, it is crucial to acknowledge that combinations of SNPs that are not significantly correlated with ADs could still contribute to the genetic predisposition for ADs (40). Furthermore, the Steiger test was utilized to ascertain whether reverse causality had an impact on the observed causality. This test evaluates whether the selected SNPs explain the variance in ADs more effectively than the identified metabolites. In cases where the collective SNPs were determined to not pose a genetic risk for ADs in comparison to the metabolites, the results indicated the absence of bias in causal inference (Steiger *P*< 0.05).

### Meta-analysis and metabolic pathway analysis

The robustness of the candidate metabolites, identified based on the aforementioned criteria, was thoroughly assessed by replicating the IVW analysis in an additional six AD cohorts. In essence, the initial analysis employed GWAS datasets designated as discovery, while supplementary GWAS datasets were utilized for the replication analysis. A meta-analysis of two MR analyses was conducted to determine the serum metabolites with causal effects on ADs. This meta-analysis employed a random-effects IVW model using the meta package (41).

To identify the final candidates from the additional GWAS data of ADs, significant associations (*P*_IVW_< 0.05) were evaluated through replication analysis and meta-analysis. MetaboAnalyst 5.0 (https://www.metaboanalyst.ca/) was employed for the metabolic pathway analysis of known metabolites. Two databases, namely the KEGG database and the Small Molecule Pathway Database (SMPDB), were utilized in this study. The significance threshold value for pathway analysis was set at 0.10.

### Statistical analysis

The statistical analysis was conducted using R4.2.2 software, and the MR analysis was primarily performed using the TwoSampleMR (42) package. Additionally, error detection rate (false discovery rate; FDR) correction was employed to mitigate false positives in multiple tests. A given metabolite’s estimated causal effect was considered statistically significant when it had an FDR< 0.05, it was considered statistically significant. The data and source code can be downloaded from https://github.com/wewen1996/Two-sample-Mendelian-Randomization-and-Meta-Analysis.

## Results

### Selection of instrumental variables

A total of 486 metabolites were selected, with the number of instrumental variables (IVs) ranging from 3 to 485, and a median of 27 (**Supplementary Table 4**). These IVs accounted for a variance in their corresponding metabolites ranging from 0.0082% to 188.8405%. The MR-PRESSO global test did not provide any evidence of pleiotropic effects (*P* > 0.05). Importantly, all the minimum F-statistics for the validity test exceeded 10, ranging from 17.64 to 21.00 (**Supplementary Table 4**). This indicates that weak instrument bias was unlikely to have occurred.

### Causal effects of metabolites on autoimmune diseases

To gain a more comprehensive understanding of metabolic changes, this analysis excluded 177 unidentified metabolites and focused on the 309 metabolites with known structures and functions, estimating the causal relationship between these metabolites and six AD phenotypes using the selected IVs.

**Figure 2:**
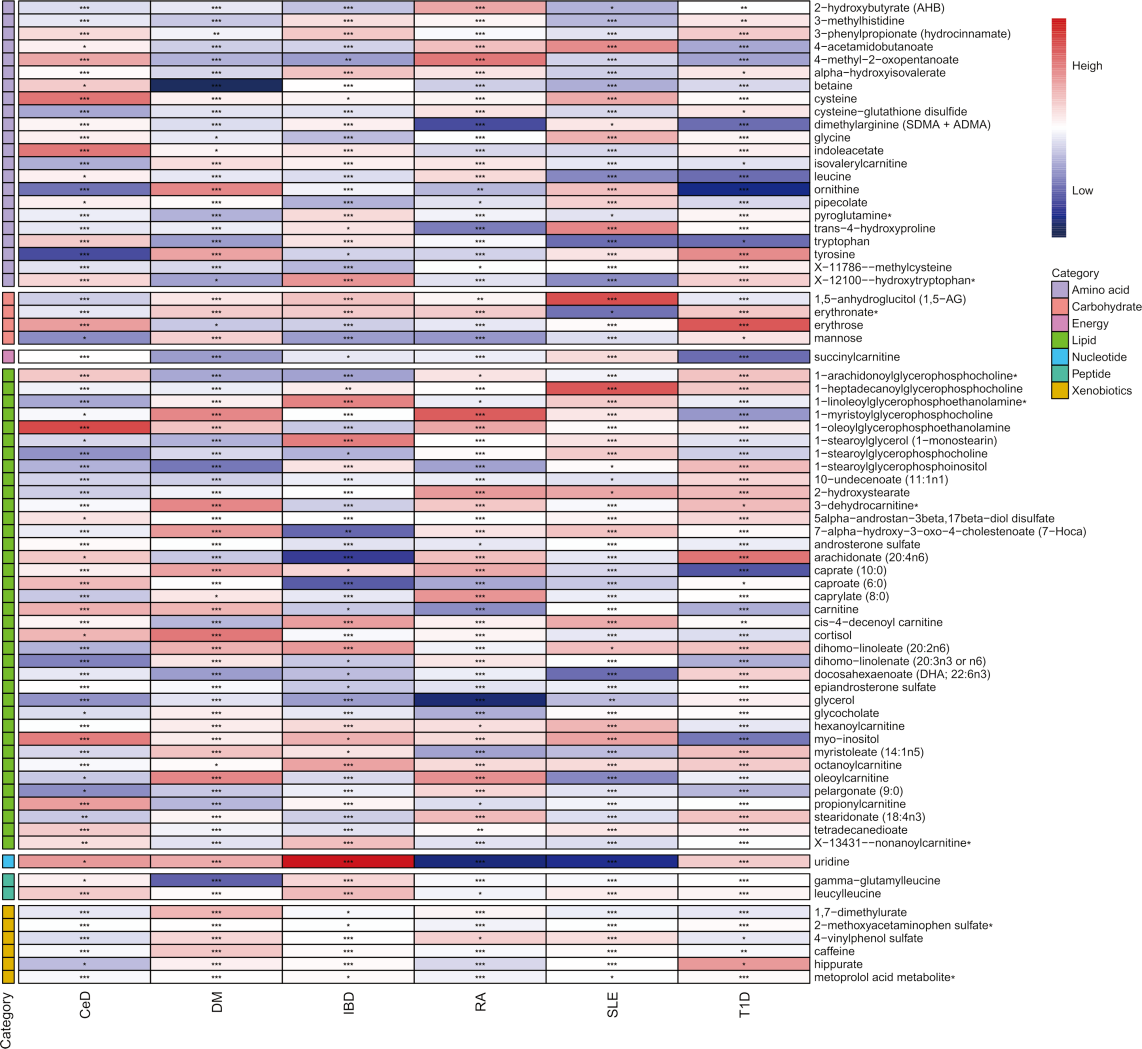
Heatmap of mendelian randomized association effect values for known metabolites with significant differences in phenotypic risk for six autoimmune diseases (from fixed effects IVW analysis). (^*^: *P*<0.05; ^**^: *P*<0.01; ^***^: *P*<0.001).

In the discovery phase, the first one or two serum metabolites most associated with the risk of CeD, DM, IBD, SLE, RA, SLE, and T1D were screened using six MR methods. Assumption checks were conducted for all 309 regulators to determine the most suitable analytical tools, with the IVW method being selected as the primary approach due to the absence of heterogeneity and weak instruments. Following multiple test adjustments using the false discovery rate (FDR) *P*-value threshold, eight metabolic exposures were identified as statistically significant at a threshold of *P*_FDR_< 0.05 (**Figure 2**). It is noteworthy that exposure factors associated with CeD and RA, represented by SNPs 3 and 6, were incorporated into the results (**Table 2**). Despite the limited number of SNPs, which may lead to reduced statistical power or the presence of weak instrumental variables, subsequent statistical analysis confirmed that these two exposure factors met the criteria for strong instrumental variables, with their statistical power values aligning with the standard requirements for exposure factors. The statistical robustness of our findings aligns with the standard requirements for exposure factors, resulting in the retention of the results. These associations encompassed five metabolites from the lipid pathways, two from the lipid metabolism pathways, one from the amino acid pathways, and two from the xenobiotic pathways. Notably, our results revealed a substantial causal association between a heightened susceptibility to CeD and an elevated level of 1-oleoylglycerophosphoethanolamine (odds ratio [OR] = 11.271, 95% confidence intervals [CI]: 2.053-61.882, *P* = 0.005, *P*_FDR_ = 0.032). Furthermore, we observed that the onset of DM could elevate the level of betaine (OR = 0.014, 95% CI: 0.001-0.318, *P* = 0.007, *P*_FDR_ = 0.042). Additionally, 1-arachidonoylglycerophosphoethanolamine (OR = 0.411, 95% CI: 0.264-0.642, *P* = 9.034×10^-5^, *P*_FDR_ = 5.420×10^-4^) and arachidonate (20:4n6) (OR = 0.352, 95% CI: 0.195-0.635, *P* = 5.132×10^-4^, *P*_FDR_ = 0.002) were found to be elevated in IBD patients. The causal effect of RA on 1-myristoylglycerophosphocholine was estimated at 1.009 (95% CI: 1.004-1.014, *P* = 0.001, *P*_FDR_ = 0.003), while glycerol was estimated at 0.991 (OR = 0.991, 95% CI: 0.986-0.997, *P* = 0.005, *P*_FDR_ = 0.031). A positive association was observed between 2-methoxyacetaminophen sulfate and SLE (OR = 0.945, 95% CI: 0.920-0.971, *P* = 4.008×10^-5^, *P*_FDR_ = 1.477×10^-4^). Conversely, glycerol 2-phosphate exhibited a negative association with T1D (OR = 3.382, 95% CI: 1.897-6.027, *P* = 3.599×10^-5^, *P*_FDR_ = 2.159×10^-4^). It is important to note that the values of OR of 1-myristoylglycerophosphocholine, glycerol, and 2-methoxyacetaminophen sulfate are very close to 1, implying a limited clinical impact despite their statistical significance. This discrepancy can potentially be attributed to substantial variation in the independent variable. Therefore, it is imperative to conduct further *in vivo* investigations to ascertain the clinical relevance of these three exposure factors.

**Table 2:** Significant MR analysis results in the discovery samples.

Methods	Outcome	Exposure	Category	No. SNP	OR	95% CI	*P*	*P*_FDR_
IVW	CeD	1-oleoylglycerophosphoethanolamine	Lipid	3	11.271	2.053-61.882	**0.005**	**0.032**
Weighted Median					10.380	1.255-85.843	0.030	0.060
MR-Egger					1.076	0.000-3.156	0.684	0.684
Simple Mode					21.886	1.015-471.939	0.144	0.215
Weighted Mode					7.261	0.516-102.194	0.238	0.286
MR-PRESSO					11.271	1.393-91.160	0.023	0.060
IVW	DM	betaine	Amino acid	22	0.014	0.001-0.318	**0.007**	**0.042**
Weighted Median					0.119	0.002-0.456	0.028	0.056
MR-Egger					0.002	0.000-3.585	0.121	0.242
Simple Mode					0.282	0.000-466.990	0.741	0.741
Weighted Mode					0.119	0.000-121.643	0.553	0.664
MR-PRESSO					0.014	0.000-0.422	0.014	0.042
IVW	IBD	1-arachidonoylglycerophosphoethanolamine	Lipid	26	0.411	0.264-0.642	**9.034×10^-5^**	**5.420×10^-4^**
Weighted Median					0.352	0.180-0.686	0.002	0.004
MR-Egger					0.143	0.044-0.465	0.004	0.004
Simple Mode					0.598	0.160-2.226	0.450	0.450
Weighted Mode					0.207	0.080-0.535	0.003	0.004
MR-PRESSO					0.411	0.253-0.669	3.511×10^-4^	0.001
IVW	IBD	arachidonate (20:4n6)	Lipid	20	0.352	0.195-0.635	**5.132×10^-4^**	**0.002**
Weighted Median					0.285	0.148-0.549	1.750×10^-4^	0.001
MR-Egger					0.216	0.081-0.579	0.007	0.008
Simple Mode					0.596	0.124-2.856	0.525	0.525
Weighted Mode					0.255	0.128-0.509	0.001	0.002
MR-PRESSO					0.352	0.179-0.693	0.003	0.004
IVW	RA	1-myristoylglycerophosphocholine	Lipid	6	1.009	1.004-1.014	**0.001**	**0.003**
Weighted Median					1.010	1.003-1.017	0.005	0.010
MR-Egger					1.013	1.000-1.026	0.129	0.129
Simple Mode					1.010	1.000-1.020	0.105	0.126
Weighted Mode					1.010	1.002-1.018	0.050	0.075
MR-PRESSO					1.009	1.004-1.014	0.001	0.003
IVW	RA	glycerol	Lipid	18	0.991	0.986-0.997	**0.005**	**0.031**
Weighted Median					0.993	0.986-1.000	0.048	0.096
MR-Egger					1.000	0.988-1.012	0.972	0.972
Simple Mode					0.996	0.985-1.007	0.451	0.542
Weighted Mode					0.995	0.987-1.002	0.166	0.249
MR-PRESSO					0.991	0.985-0.998	0.012	0.037
IVW	SLE	2-methoxyacetaminophen sulfate	Xenobiotics	97	0.945	0.920-0.971	**4.008×10^-5^**	**1.477×10^-4^**
Weighted Median					0.943	0.907-0.980	0.003	0.006
MR-Egger					0.921	0.853-0.995	0.038	0.056
Simple Mode					0.903	0.811-1.006	0.066	0.079
Weighted Mode					0.935	0.862-1.014	0.104	0.104
MR-PRESSO					0.945	0.920-0.971	4.924×10^-5^	1.477×10^-4^
IVW	T1D	glycerol 2-phosphate	Xenobiotics	21	3.382	1.897-6.027	**3.599×10^-5^**	**2.159×10^-4^**
Weighted Median					1.980	0.894-4.382	0.092	0.184
MR-Egger					1.226	0.410-3.668	0.718	0.718
Simple Mode					1.591	0.412-6.144	0.505	0.607
Weighted Mode					1.801	0.683-4.749	0.244	0.366
MR-PRESSO					3.382	1.757-6.509	2.654×10^-4^	0.001

The bold font is the P-value of IVW algorithm, the main analysis method used in this study, and the P-value after FDR correction.

### Sensitivity analyses

**Figure 3:**
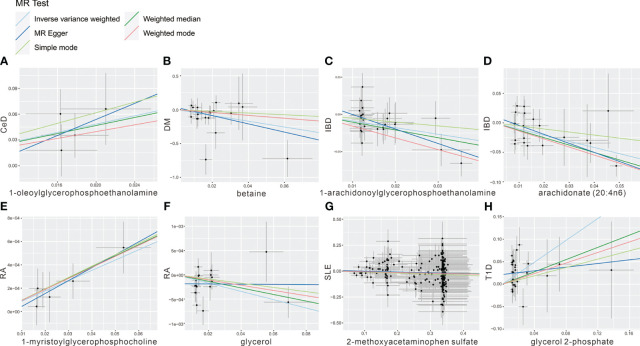
Scatter plot showing the genetic associations of seven metabolites on the risk of 6 AD phenotypes. **(A)** 1-oleoylglycerophosphoethanolamine on CeD, **(B)** betaine on DM, **(C)** 1-arachidonoylglycerophosphoethanolamine on IBD, **(D)** arachidonate (20:4n6) on IBD, **(E)** 1-myristoylglycerophosphocholine on RA, **(F)** glycerol on RA, **(G)** 2-methoxyacetaminophen sulfate on SLE, **(H)** glycerol 2-phosphate on T1D.

**Figure 4:**
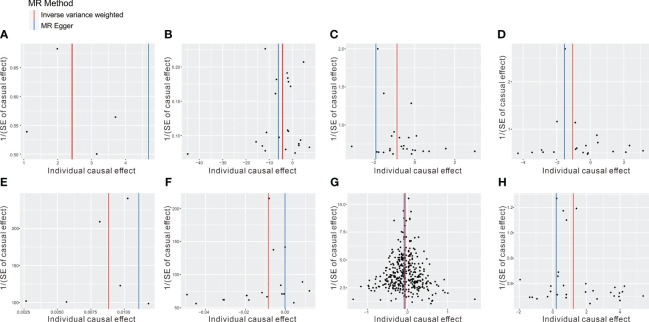
The funnel plot represents IVs for each significant causal association between metabolites and OA phenotypes. **(A)** 1-oleoylglycerophosphoethanolamine on CeD, **(B)** betaine on DM, **(C)** 1-arachidonoylglycerophosphoethanolamine on IBD, **(D)** arachidonate (20:4n6) on IBD, **(E)** 1-myristoylglycerophosphocholine on RA, **(F)** glycerol on RA, **(G)** 2-methoxyacetaminophen sulfate on SLE, **(H)** glycerol 2-phosphate on T1D.

To mitigate the horizontal pleiotropy of MR estimates, sensitivity analyses were conducted. A series of six tests were implemented for metabolites associated with multiple SNPs54t, including fixed-/random-effects IVW test, weighted median method, and MR-Egger regression test. The results of these sensitivity analyses for eight metabolites and their causal relationships with ADs are detailed in **Table 2**, demonstrating statistically significant findings. Notably, robust causality was frequently observed to exhibit statistical significance in two additional MR tests (*P*< 0.05), specifically the weighted median test and the MR-PRESSO test. All eight pairs of associations are considered robust (**Table 2**). Furthermore, an evaluation for potential horizontal pleiotropy in all these associations was undertaken using the MR-Egger intercept term and the MR-PRESSO global test (**Supplementary Table 3**). Heterogeneity among SNPs related to each metabolite was assessed using Cochrane’s Q test. In instances where heterogeneity was detected (*P*< 0.05), the random-effects IVW test was employed to offer a more conservative yet robust estimate. Additionally, scatter plots (**Figure 3**) and funnel plots (**Figure 4**) were utilized to rule out potential outliers and horizontal pleiotropy for all identified metabolites. Furthermore, the LOO analysis did not reveal substantial variation in estimates of causality between the eight metabolites and ADs, suggesting that none of the identified causal relationships were influenced by any single instrumental variable (**Supplementary Figure 1**).

Moreover, the significant metabolites identified in the initial discovery stage were successfully replicated in independent replication datasets. The replication MR analysis followed the same rigorous methodology as applied in the discovery samples, ensuring consistency and reliability in the findings.

### Replication and meta-analysis

**Figure 5:**
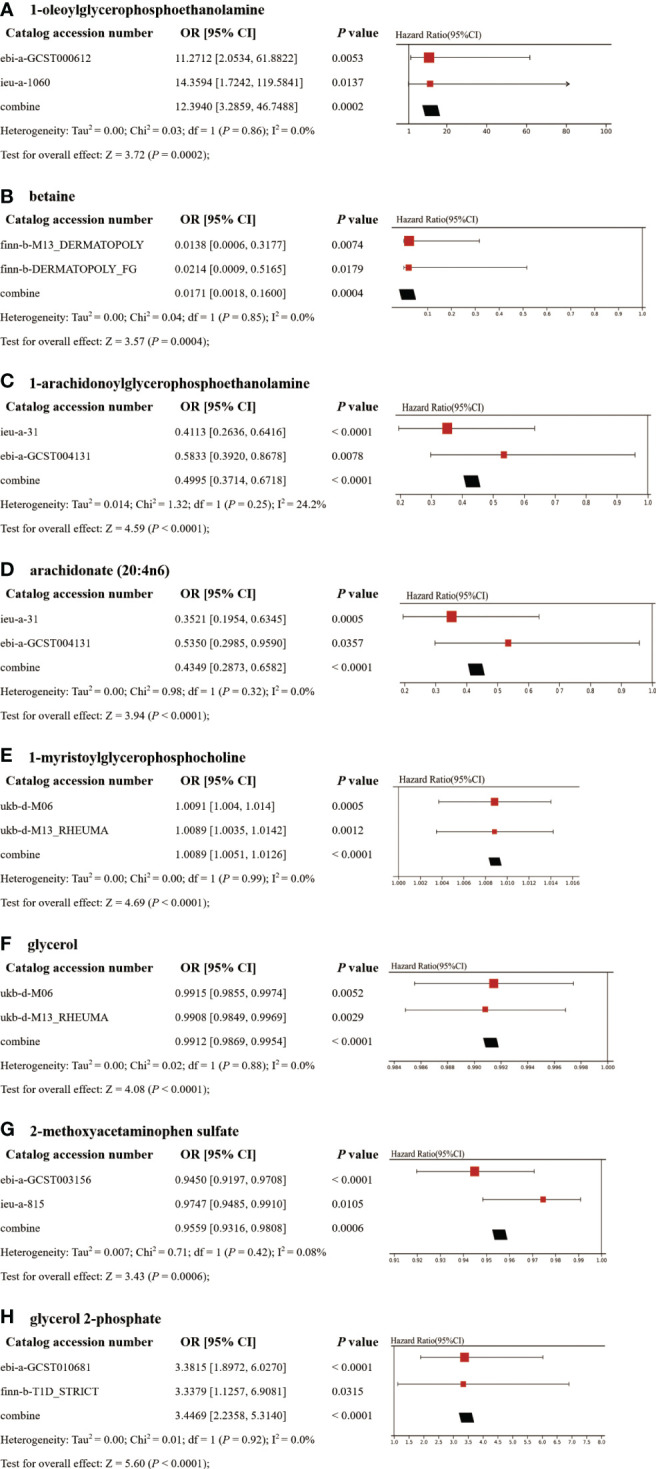
Meta-analysis of significantly associated (IVW derived P< 0.05) between metabolites and CeD **(A)**, DM **(B)**, IBD **(C–D)**, RA **(E–F)**, SLE **(G)**, and T1D **(H)**. 95% CI, 95% confidence interval; OR, odds ratio.

To enhance the robustness of the estimates, validated metabolites showing significant causal links with ADs were subjected to validation in independent replication samples (**Supplementary Table 2**). As anticipated, similar patterns were observed for the identified metabolites in replicated GWAS data for ADs. Notably, beyond SLE and T1D, statistical significance persisted for other AD-related metabolites even following FDR correction (*P*< 0.05). The meta-analysis further confirmed the impact of eight blood metabolites on ADs (**Figure 5**). Specifically, genetic predisposition for elevated levels of betaine (OR 0.02, 95% CI: 0.002-0.16, *P* = 0.0004), 1-arachidonoylglycerophosphoethanolamine (OR 0.50, 95% CI: 0.37-0.67, *P*< 0.0001), arachidonate (20:4n6) (OR 0.43, 95% CI: 0.29-0.66, *P*< 0.0001), glycerol (OR 0.99, 95% CI: 0.987-0.995, *P*< 0.0001), and 2-methoxyacetaminophen sulfate (OR 0.96, 95% CI: 0.93-0.98, *P* = 0.0006) was associated with reduced susceptibility to ADs. Conversely, genetic predisposition for higher levels of 1-oleoylglycerophosphoethanolamine (OR 12.39, 95% CI: 3.29-46.75, *P* = 0.0002), 1-myristoylglycerophosphocholine (OR 1.0089, 95% CI: 1.0051-1.0126, *P*< 0.0001), and glycerol 2-phosphate (OR 3.45, 95% CI: 2.24-5.31, *P*< 0.0001) was associated with increased susceptibility to ADs. Importantly, the Steiger test results confirmed the accuracy of the selected IVs, with a *P*-value significantly below 0.05.

### Metabolic pathway analysis

Although shared causal metabolites were identified across the six ADs, it is noteworthy that five out of the eight significantly associated metabolites were lipid compounds. This finding suggests the potential role of lipids as key components among plasma metabolites associated with ADs. Analysis based on the eight identified metabolites revealed enrichment of six metabolic pathways in the KEGG and SMPDB databases, which could play a role in the pathogenesis of ADs (**Supplementary Tables 5, 6**). Within the KEGG database, these metabolites were primarily enriched in pathways such as glycerolipid metabolism, galactose metabolism, glycine, serine, and threonine metabolism, biosynthesis of unsaturated fatty acids, glycerophospholipid metabolism, and arachidonic acid pathways. Particularly noteworthy was the significant enrichment of glycerolipid metabolism pathway with a P-value below 0.05, indicating its potential relevance to common ADs. In contrast, analysis using the SMPDB database showed enrichment of metabolites in pathways including betaine, glycerolipid, galactose, methionine, glycine, serine, and arachidonic acid metabolism.

## Discussion

The early-stage symptoms of ADs often go unnoticed, with the detection of antibodies being relatively costly (43). As a result, patients are frequently diagnosed when they have already reached an advanced and irreversible stage of the disease. Early screening for ADs can serve as a proactive measure, alerting individuals at risk to consider lifestyle adjustments and prioritize efforts to prevent disease progression (44). Serum metabolites, which are relatively easy to obtain and can be detected using less invasive methods, can serve as biomarkers for the early screening of ADs. However, prior MR studies examining the relationship between ADs and serum metabolites were limited to specific GWAS samples, lacking replication and integrated analyses (45). Therefore, in order to obtain more robust results, MR and meta-analyses were conducted to evaluate the association between serum metabolites and ADs.

Blood, recognized as a reliable source for assessing metabolite levels, contains numerous detectable metabolites and can be easily obtained in substantial sample sizes, facilitating the screening of circulating risk markers for AD. This two-sample MR study represents a significant advancement in elucidating the causal relationship between 73 metabolites and six AD phenotypes. Eight of these metabolites exhibited strong associations that persisted even after correction for multiple testing, underscoring their promise as dependable biomarkers or targets for therapeutic intervention. These results were substantiated through sensitivity analyses, bolstering their reliability. This study provides further analytical perspectives on the impact of gene-environment interactions in the development of ADs. Delving into the functional implications of these metabolites within metabolic pathways may pave the way for future precision medicine strategies. Furthermore, this study in question affirmed the presence of an AD-specific metabolic profile and identified critical metabolites and metabolic pathways causally associated with the development of ADs.

Over the past decade, numerous studies have underscored the close relationship between AD onset and human metabolism. This association is evident not only through its co-occurrence with various symptoms of metabolic disorders but also due to the emergence of metabolite-related dysfunctions within immune cells in metabolomics studies (46–49). Furthermore, research has demonstrated that intracellular metabolism can significantly impact the state of immune cells, suggesting potential avenues for developing novel therapeutic targets to counter dysfunctional antigen-induced immune responses in ADs, which are closely linked to blood metabolite concentrations. Published research has illuminated the role of L-Arginine in regulating T cell metabolism, thereby influencing T cell differentiation and outcomes (50). Additionally, studies have revealed the protective function of Selenium-GPX4 on follicular helper T cells (51). Notably, in the study, five out of the eight metabolites identified as causally associated with the pathogenesis of ADs belong to the category of lipid metabolites, including 1-oleoylglycerophosphoethanolamine, 1-arachidonoylglycerophosphoethanolamine, arachidonate (20:4n6), 1-myristoylglycerophosphocholine, and glycerol. Circulating lipids play a significant role in immune cell function. Lipid uptake or efflux influences cellular lipid burden and function, which is particularly notable in autoimmunity, where dyslipidemia and cardiovascular complications are common. The metabolism of lipids is crucial in a range of ADs (52–54). Elevations in both cell membrane glycosphingolipids and cholesterol are associated with heightened T cell and B cell receptor signaling, leading to activation and inflammation. Immune cells generate lipoxins, resolvins, and protectins through the enzymatic conversion of omega-3 fatty acids, playing a role in resolving inflammation and restoring tissue homeostasis. Their levels are linked to reduced joint pain in patients with RA and are decreased in experimental models of RA with persistent joint inflammation (52).

Lysophospholipids, characterized by a single fatty acid, play a role in regulating the five primary indicators of inflammation: *rubor* (redness), *tumor* (swelling), *calor* (fever), *dolor* (pain), and *functio laesa* (loss of function) (55). Currently, research mainly focuses on lysophospholipic acid (LPA) and sphingosine 1-phosphate (S1P) among lysophospholipids. Advances in lysophospholipid research have led to the development of novel treatment strategies for ADs, with numerous therapies currently in the early stages of development for various conditions, including fibrotic disorders, vascular diseases, and cancer. Among the two types of lysophospholipids, our research results indicate that 1-oleoylglycerophosphoethanolamine plays a protective role in CeD, while 1-myristoylglycerophosphocholine provides protection in RA. Several observational and experimental studies have reported the involvement of lysophospholipids in regulating immune cells. Lysophospholipids are involved in the resolution processes that counteract the protective mechanisms of normal inflammation. For example, lysophospholipids can influence the activity of traditional regulators of vascular permeability, such as histamine, serotonin, and bradykinin, to positively or negatively control vasodilation, vasoconstriction, and vascular leakage. G-protein-coupled S1PRs expressed by endothelial cells mediate vasodilation and inhibit vascular leakage by promoting the assembly of adherens junctions. On the other hand, vascular smooth muscle expresses Gq- and G12/13-coupled S1PRs and LPARs, which induce vasoconstriction and promote vascular leakage under inflammatory conditions. Additionally, lysophospholipids also regulate hematopoietic and immune cells during inflammation. Platelet aggregation, neutrophil phagocytosis, macrophage fate switching, innate immunity, natural killer cell release into circulation, and the trafficking and tissue residence of adaptive (T and B) cells are all regulated by lysophospholipid signaling via G protein-coupled receptors (GPCRs), impacting inflammatory and resolution responses (55). Although the two metabolites identified in this study have not been extensively investigated, they hold great research potential considering the function of relatively mature lysophospholipids in the immune process.

Moreover, our study identified two arachidonic acids, 1-arachidonoylglycerophosphoethanolamine and arachidonate (20:4n6), which showed a significant causal association with the onset and progression of IBD, highlighting their potential role in promoting susceptibility to this condition. Arachidonic acid is converted into active metabolites by enzymes such as cyclooxygenase (COX), lipoxygenase, and cytochrome P450 (CYP). Downstream eicosanoid signaling can directly impact the metabolism of immune cell subsets by regulating PPAR, controlling the liver X receptor (LXR), and mediating anti-inflammatory effects. Prostaglandin signaling can either stimulate or inhibit the anti-inflammatory ability of PPARγ to counteract NF-κB in various immune cells (52). Additionally, eicosanoids produced by arachidonic acid metabolism are crucial mediators of inflammation, and some of their metabolic network proteins have become important targets for anti-inflammatory drug design (56). Among these metabolites, prostaglandin E2 (PGE2) is the most widely studied in IBD, with elevated levels observed in individuals with active ulcerative colitis (57). Recent research has highlighted the involvement of the 12-lipoxygenase pathway, one of the metabolic pathways of AA, in intestinal inflammation (58). Although the pro-inflammatory effect of arachidonic acid in the pathogenesis of IBD is established, the clinical utilization of non-steroidal anti-inflammatory drugs (NSAIDs) to block its metabolites suggests that its mechanism of action needs further investigation (59). Moreover, while 1-arachidonoylglycerophosphoethanolamine is associated with dermatologic diseases, and arachidonate (20:4n6) is linked to various ADs, their potential role in IBD has not been previously documented. Given the close relationship between lipid metabolites and the development of IBD, further research on these two metabolites may represent a promising avenue for future investigation.

Another significant finding from our study is the detrimental effect of 2-methoxyacetaminophen sulfate in SLE, while glycerol 2-phosphate demonstrates a protective effect in T1D. It is noteworthy that both of these metabolites are classified as xenobiotics, which are foreign substances not naturally present in the body. Exogenous metabolism, as a crucial pathway in the body, plays a vital role in regulating and detoxifying such chemicals to prevent potential harm caused by environmental substances. Specifically, 2-methoxyacetaminophen belongs to the class of acetamides and is a paracetamol sulfate derivative with a methoxy group substitution at position 3 (60). On the other hand, glycerol 2-phosphate is a bacterial metabolite produced during a metabolic reaction within Escherichia coli (61). The discovery of xenobiotics further emphasizes the intricate relationship between ADs, heredity, and the environment. It highlights the need for future research on ADs to move beyond solely focusing on individual genetic or environmental factors in isolation. Instead, it suggests that the pathogenesis of ADs likely arises from complex interactions between genes and the environment.

Furthermore, the study findings indicated that betaine serves as a pathogenic risk factor for DM. Betaine is acknowledged for its essential roles as an osmoprotectant and methyl group donor in physiological processes. Numerous pieces of evidence have highlighted the anti-inflammatory properties of betaine in various conditions, including obesity, diabetes, cancer, and Alzheimer’s disease (62). Surprisingly, limited research has explored the impact of betaine on DM specifically. Given the well-known anti-inflammatory attributes of betaine, there is a clear necessity for further investigation to uncover the precise mechanisms through which betaine influences the development of DM.

In this study, we identified metabolic pathways that play a causal role in the development of ADs. Some of these pathways have been extensively studied experimentally and are well-documented to contribute to the pathogenesis of ADs. Moreover, our analysis of KEGG and SMPDB data revealed a robust association between the pathogenesis of ADs and glycerolipid metabolism, as well as galactose metabolism pathways. Furthermore, it was observed that the differentiation and function of immune cells involved in the inflammatory response are intricately linked to the process of glycerol and lactose metabolism (63–65). These findings suggest that targeting this interconnected metabolic network could offer a promising approach for intervening in the autoimmune state and ultimately treating ADs.

The present study offers several notable advantages. Firstly, its main strength lies in the broad range of genetic variables considered to investigate the association between blood metabolites and various phenotypes of ADs. Specifically, this study encompassed a comprehensive panel of 486 metabolites, excluding those yet to be identified. Additionally, genetic variables for each AD were sourced from two separate datasets, and a meta-analysis was conducted to combine the analytical outcomes from these dual databases. This approach enabled a relatively comprehensive and systematic analysis of the metabolic profile associated with the development of ADs. Secondly, the utilization of the MR Design in this study significantly mitigated issues related to reverse causality and residual confounding factors. The extensive sensitivity analysis effectively accounted for potential influences of variable polymorphisms. Consequently, the inference of a causal relationship between metabolites and the risk of ADs in this study is considered robust.

However, the study also has certain limitations. Firstly, the MR analysis was based on blood metabolomics data, and although it identified some serum metabolites with a causal relationship to ADs, further clinical empirical studies are needed to identify more promising biomarkers and potential drug targets. Secondly, the metabolite data predominantly originated from European populations, limiting the generalizability of these findings to different ethnic groups. Thirdly, while this study encompassed a relatively comprehensive metabolite profile, the functions and mechanisms of certain metabolites in the context of disease remain incompletely understood. This limitation affects the full interpretation of the results of this MR analysis.

## Conclusion

In summary, this study presents a systematic MR and meta-analysis utilizing GWAS data to evaluate the causal relationship between serum metabolites and various AD phenotypes. It offers preliminary evidence for the impact of circulatory metabolic disorders on AD risk. By employing the IVW method and conducting multiple sensitivity analyses, robust causal relationships were established between eight metabolites and six AD phenotypes. Furthermore, the analysis of metabolic pathways revealed that these eight metabolites are enriched in six significant metabolic pathways. Our findings suggest that elevated levels of lipid metabolites may contribute to the development of ADs. This implies that specific metabolites and genetic susceptibilities may serve as biomarkers for the risk of ADs, potentially enabling earlier diagnosis and more effective treatment options.

## Data availability statement

The original contributions presented in the study are included in the article/**Supplementary Material**. Further inquiries can be directed to the corresponding authors.

## Author contributions

WW: Conceptualization, Data curation, Software, Writing – original draft, Writing – review & editing. MH: Data curation, Investigation, Methodology, Writing – original draft. WG: Investigation, Methodology, Supervision, Writing – review & editing. JF: Data curation, Supervision, Writing – review & editing. XZ: Methodology, Software, Writing – review & editing. CL: Formal analysis, Supervision, Validation, Writing – review & editing. LW: Investigation, Software, Supervision, Writing – original draft, Writing – review & editing.
