# Supplementary figures and images for: Identifying serum metabolite biomarkers for autoimmune diseases: a two-sample mendelian randomization and meta-analysis
Source: Front Immunol. 2024 Apr 15;15:1300457. doi: 10.3389/fimmu.2024.1300457 (PMC11056515; doi:10.3389/fimmu.2024.1300457)

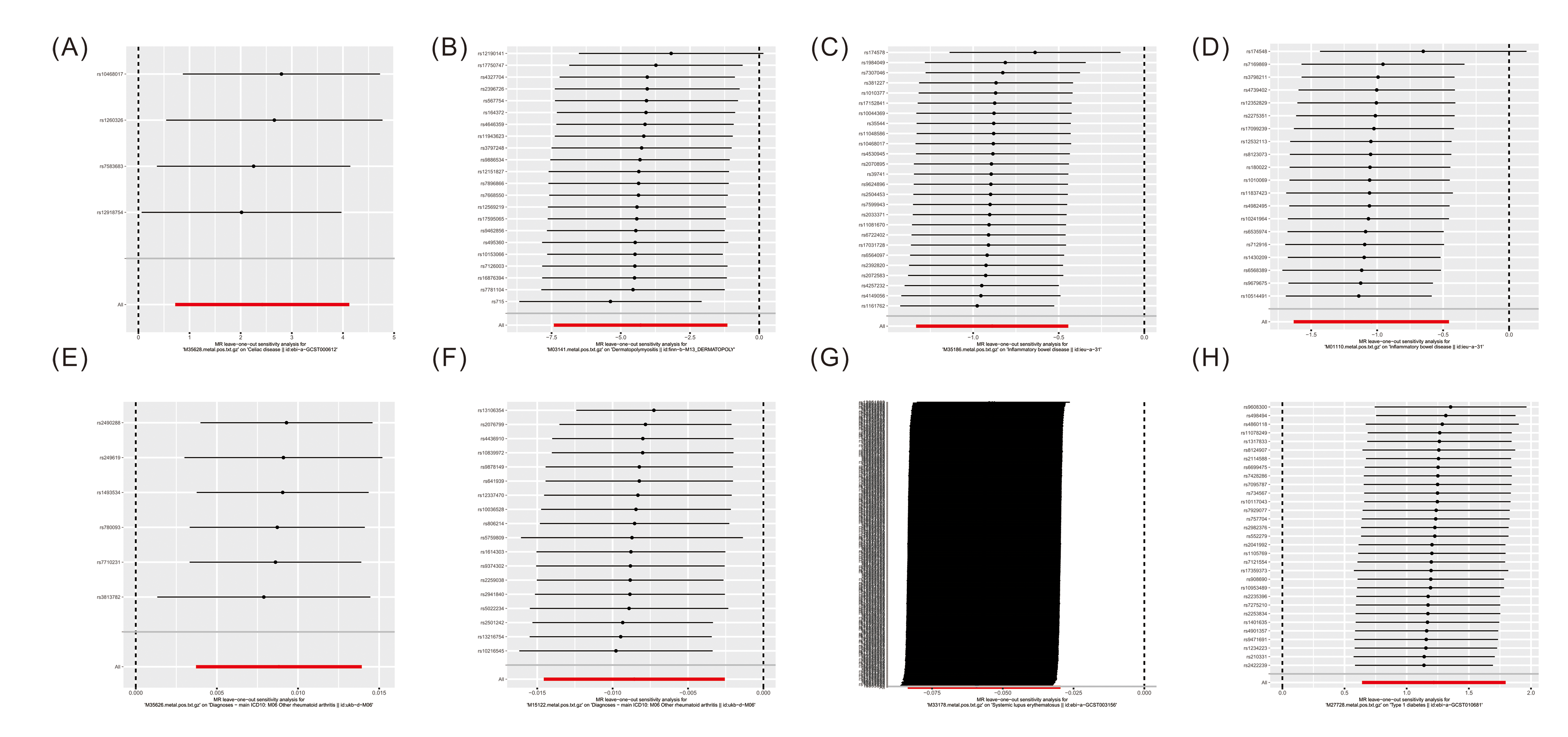

Supplement: Supplementary file 2 [file Image_1.tif]
